# Heart Rate Responses and Training Load During Nonspecific and Specific Aerobic Training in Adolescent Taekwondo Athletes

**DOI:** 10.2478/v10078-011-0040-y

**Published:** 2011-10-04

**Authors:** Monoem Haddad, Anis Chaouachi, Del P. Wong, Carlo Castagna, Karim Chamari

**Affiliations:** 1 Tunisian Research Laboratory “Sports Performance Optimisation” National Center of Medicine and Science in Sports (CNMSS), Tunis, Tunisia.; 2 Department of Health and Physical Education, The Hong Kong Institute of Education, Hong Kong; 3 School of Sport and Exercise Sciences, Faculty of Medicine and Surgery, University of Rome Tor Vergata, Rome, Italy

**Keywords:** interval training, martial arts, youth, physical integrated training

## Abstract

The efficacy of replacing generic running with Taekwondo (TKD) specific technical skills during interval training at an intensity corresponding to 90–95% of maximum heart rate (HR_max_) has not yet been demonstrated. Therefore, the purpose of this study was to compare the HR responses and perceived exertion between controlled running and high-intensity TKD technical interval training in adolescent TKD athletes. Eighteen adolescent, male TKD athletes performed short-duration interval running and TKD specific technical skills (i.e. 10–20 [10-s of exercise interspersed with 20 s of passive recovery]) in a counterbalanced design. In both training methods, HR was measured and expressed as the percentage of HR reserve (%HR_res_). Rating of perceived exertion (RPE, Borg’s category rating-10 scale), Banister’s training impulse (TRIMP) and Edwards’ training load (TL) were used to quantify the internal training load. Recorded cardiovascular responses expressed in %HR_res_ in the two training methods were not significantly different (p > 0.05). Furthermore, the two training methods induced similar training loads as calculated by Banister and Edwards’ methods. Perceived exertion ranged between “hard” and “very hard” during all interval training sessions. These findings showed that performing repeated TKD specific skills increased HR to the same level, and were perceived as producing the same training intensity as did short-duration interval running in adolescent TKD athletes. Therefore, using specific TKD kicking exercises in high-intensity interval training can be applied to bring more variety during training, mixing physical and technical aspects of the sport, while reaching the same intensity as interval running.

## Introduction

The quality of the development of technical skills throughout a Taekwondo (TKD) competitive season depends on the specific functional and physical preparation for task requirements. TKD competition is considered as an intermittent exercise with alternative aerobic and anaerobic sequences (Bouhlel et al., 2006).Bouhlel et al. (2006) showed that TKD requires high levels of both aerobic and anaerobic physical fitness. Maximum oxygen uptake (VO2 max) values measured and correlation between HR responses and blood lactate concentrations measured during the 3-min specific exercise in TKD competition showed the importance of aerobic metabolism for elite TKD athletes (Bouhlel et al., 2006). Matsushigue et al. (2009) recommended that coaches should include high-intensity interval training to prepare elite TKD athletes to cope with the metabolic and physiological demands of competition. To the best of our knowledge, there are only few studies available that have examined training programs of TKD athletes.

The training effects of high-intensity interval running have been reported in different types of athletes (Billat et al., 1999). Most of these studies have shown improved VO2 max, an increase in oxygen transport capacity, a higher aerobic contribution to energy expenditure (Balsom et al., 1994), and consequently, reduced fatigue through glycogen sparing and better buffering of muscle pH as compared with continuous running. Balsom et al. (1993) showed that short-duration, high-intensity, intermittent training allowed limited lactate production and increased creatine phosphate metabolism during intermittent exercise. Creatine phosphate and muscle glycogen were described as the most important energy substrates for this type of training (Balsom et al., 1993). In a previous study designed for adolescents, Baquet et al. (2001) showed that 10 seconds of high-intensity intermittent running interval at 100–120% of maximal aerobic speed (i.e. the lowest speed that elicits VO2 max for conventional runs) could improve aerobic fitness. Dawson et al. (1998) demonstrated that 6 weeks of short sprint training at 90–100% of maximum speed could improve endurance, sprint and repeated sprint ability in fit subjects. Also, Obert et al. (2001) and Baquet et al. (2004) emphasized the effectiveness of an aerobic training program to improve maximal power during short-term exercise in children. These previous studies have shown that this type of a training method has the potential to improve the athletes’ endurance at various ages.

However, running based training is far from TKD-specific activities. Indeed, during competition, technical gestures are performed at high intensity (Bouhlel et al., 2006; Matsushigue et al., 2009), and therefore TKD sessions should include technical and tactical aspects required in competition. Previous studies have focused on the intensity of TKD exercises in short duration sequences lasting from 10 s to 3 min (Bouhlel et al., 2006) and in various conventional activities in TKD (Bridge et al., 2007; Matsushigue et al., 2009). The possibility of performing high-intensity, TKD specific interval training was proposed by Matsushigue et al. (2009). However, to the authors’ knowledge, there are currently no studies that have focused on cardiovascular responses during high-intensity TKD specific training. Therefore, the purpose of the present study was to compare HR responses and perceived exertion between running (short duration, interval runs) and TKD specific technical training (high-intensity, TKD technical interval training) in adolescent Taekwondo athletes. We postulate that it is possible to replace running interval training (short duration) with TKD specific technical skills (high-intensity, TKD technical interval training).

## Methods

### Subjects

Eighteen adolescent male TKD athletes belonging to a Tunisian National Club volunteered to participate in the study. Subjects trained three to five sessions a week for the six past years. All participants gained medical clearance from the team physician to ensure they were in good health. Parents and athletes gave their written informed consent prior to involvement in the study. The study was conducted according to the Declaration of Helsinki and the protocol was fully approved by the Ethics Committee of the National Centre of Medicine and Science in Sports of Tunis (CNMSS) before beginning of the assessments. The subjects’ physical characteristics are presented in Table 1.

### Study Design

During the week-long experimental period, two study-related sessions were implemented: short-duration interval running and TKD specific technical interval training both in the 10–20 form (10 s of exercise interspersed with 20 s of passive recovery). Athletes involved in this study were matched and randomized according to their aerobic performances into two groups and they all performed the two study-related sessions in a counterbalanced design for controlling order effects in a repeated measures design. In this design to control for order effects, groups of athletes were separated as described below and each group performed the two modalities of training in a different order. The first group practiced the nonspecific (i.e. short-duration interval running) and afterwards specific aerobic training (i.e. specific TKD technical interval training) in the morning at 10 a.m.The second group performed the specific training before the nonspecific aerobic one in the afternoon at 7 p.m. The variance due to order effects becomes a separate source of variance, making for a more powerful design. The two modalities of training were separated by 48 hours of recovery. The methodological counterbalanced design, with the respective average ambient conditions on each of the legs of the study was more detailed in Table 2. The week preceding the experimental period, which was considered as a familiarization period, was similar to the experimental week in the order in which the two study-related sessions were undertaken. Athletes were initially tested with a maximal field test (Yo-Yo Intermittent Recovery Test – level one) (Krustrup et al., 2003) in order to measure maximal running speed (Vmax) and peak heart rate (HRmax). Each athlete was verbally encouraged to give maximal effort during the entire training program. An appropriate standardized warm-up was performed before each training session.

### Procedures

#### HR Measurements and Calculations

The athletes’ HR (recorded in 5-second intervals) was continuously recorded using Polar Team System HR monitors (Polar team-system, Kempele, Finland), during each training session. The HR time-course was recorded from the beginning to the end of each training session. Resting HR was measured as the athletes lay on a bed for 10 minutes at 10:30 a.m. The resting HR value corresponded to the minimal HR observed during this 10-minute period. During the Yo-Yo IR1 field test, the highest averaged value of three consecutively recorded HR_s_ (15 seconds) was considered as HR_max_. The percentage of HR reserve (%HR_res_) was calculated for each short-duration training session by the following formula (Karvonen et al., 1957):

%HRres=(exercise mean HR−resting HR)/(HRmax−resting HR)×100

#### Field Testing

The Yo-Yo intermittent recovery (Yo-Yo IR1) field test (Krustrup et al., 2003) was performed in the same afternoon for all athletes from 6 to 7:30 p.m. in ambient conditions of 15 °C, 1018 mm Hg atmospheric pressure, and 88% relative humidity. The test was performed in a Donjon (i.e. Taekwondo gymnasium), and athletes wore a Dobok (i.e. Taekwondo uniform). Athletes were familiar with this testing procedure as it was often used to set the training pace during training sessions. Yo-Yo IR1 consists of 2×20m bouts of progressive speed shuttle-running, interspersed by 10s of active recovery, until exhaustion (Krustrup et al., 2003). This test estimated the athlete’s Vmax and allowed for the measurement of HRmax during the last 2×20m bout (Castagna et al., 2006).

#### Interval Training

The aerobic interval training consisted of 4 bouts of exercise lasting for 4 minutes with 4 minutes of active recovery in-between. The ratio 1:1 (4’:4’) allowed us to implement the sparing. Each bout consisted of short duration high-intensity interval exercise, (i.e. 10:20 (10 s of exercise interspersed with 20 s of passive recovery). The intensity corresponded to 90–95% of HRmax during running interval training. During these sessions, running distances were individualized based on the athlete’s measured Vmax and all athletes performed 100% of his Vmax during each 10 s run. During specific TKD training, Bandal Chagui or roundhouse kick techniques were used. These kicks are the most frequently used in competition (Falco et al., 2009). The roundhouse kick, a multiplanar skill, starts with the kicking leg traveling in an arc towards the front with the knee in a chambered position. The knee is extended in a snapping movement, striking the opponent with the metatarsal part of the foot extended. Following the warm-up, each athlete performed maximum repeated kicks (i.e. Bandal Chagui) during 10 s. Verbal encouragements were used to keep 90–95% of athlete’ maximal repeated kicks through the 4 bouts of specific TKD training. TKD techniques were used intermittently as during running (i.e. 10:20 [10 s of exercise interspersed with 20 s of passive recovery]).

#### Methods for Quantifying Interval Training Load (TL)

The first HR-based method of determining internal TL in the present study was the training impulse (TRIMP), described by Banister (1991). Training impulse was determined using the following formula:

$$\text{TD}\times {\text{HR}}_{\text{res}}\times \text{Y}$$

In which TD is the effective training session duration (time duration) expressed in minute and Y is a nonlinear coefficient given by the equation, Y = 0.64e^1.92x^, with e = base of the Napierian logarithms and x = HR_res_.

The HR-based method proposed by Edwards (1993) was also used to determine internal TL. This method determines internal load by measuring the product of the accumulated training duration (minutes) of five HR zones by a coefficient relative to each zone (50–60% of HRmax=1, 60–70% of HRmax=2, 70–80% of HRmax=3, 80–90% of HRmax=4, 90–100% of HRmax=5) and then summating the results.

##### Perceived Exertion

Rating of perceived exertion (RPE, Borg’s category ration-10 scale) modified from Foster et al. (2001) was also used as a measure of each interval training load. Each athlete’s RPE was collected immediately after the completion of each interval training session to ensure that the perceived effort referred to the interval training session only. All athletes who participated in this study had been familiarized with CR-10 scale for RPE before the commencement of this study.

### Statistical analysis

Values were expressed as mean ± SD. The normal distribution of the data was checked using the Kolmogorov-Smirnov test. After confirming normal distribution, two-way ANOVA with repeated measures (i.e. modalities of training vs groups) was used to evaluate the differences in %HRres, HR-based training loads and session-RPE between short-duration interval running training and specific TKD technical interval training modalities between the two groups. Oneway ANOVA with repeated measures was also used to compare the same dependents variables between the two modalities using counterbalancing analysis (i.e. the results of the same modality training were summated between the two groups). Significant within subject effects were further analyzed using paired samples t-test.

Pearson product-moment correlation coefficients were calculated to determine whether there was a significant relationship between session-RPE and HR-based training loads.

Significance was set at 5% (p ≤ 0.05). SPSS statistical software package (V.13, SPSS Inc., Chicago, IL, USA) was used for all statistical calculations.

## Results

The two-way ANOVA with repeated measures showed that there was no significant difference between running and TKD-specific interval training (F = 2.08, p > 0.05). Basing on the counterbalancing analysis, HR responses were not significantly different between running and TKD-specific interval training during training sessions (t = 1.93, p > 0.05, Table 3).

Moreover, there were no significant differences found in Banister’s TRIMP (t = 1.88, p > 0.05) and Edwards’ TL (t = 2.04, p > 0.05) in the two training modes (Table 3).

RPE values corresponded to ‘hard’ and ‘very hard’, but were not significantly different between the two training methods (t = −1.26, p > 0.05, Table 3).

No significant correlations were found between all session-RPE collected and all the HR based TL (i.e. Banister’s TRIMP [r = 0.14, p > 0.05], and Edwards’ TL [r = 0.20, p > 0.05]). During the study no injury was reported.

## Discussion

The purpose of the present study was to compare HR responses between controlled short-duration interval running and TKD specific technical interval exercises in adolescent TKD athletes. Although some studies (Balsom et al., 1993; 1994; Baquet et al., 2001) have demonstrated the effectiveness of short-duration interval running, others have suggested specific TKD technical interval training in order to improve TKD-specific aerobic endurance (Matsushigue et al., 2009). The present study showed that the TKD-specific technical interval training induced similar HR responses, perceived exertion and training loads as did short-duration interval training in adolescent TKD athletes. This result supports the hypothesis of the present study and shows that specific TKD interval training can produce similar cardiovascular stress and perceived exertion as that of short-duration interval running specifically designed to improve an athlete’s endurance. Moderating runs by specific TKD technical exercises imposes a specific activity and allows the concomitant improvement of technical skills.

The effect of replacing nonspecific physical training by specific training has been proposed for various sports activities especially in soccer (Dellal et al., 2008; Impellizzeri et al., 2006). Dellal et al. (2008) showed that some small-sided games in soccer allow the HR to increase to the same level as that measured during short-duration interval running. The integrated training method can be used to bring more variety during training, by mixing physical, technical and tactical aspects of the sport, while producing similar intensity as compared with short-duration interval running. Furthermore, the intensity of soccer-specific small-sided games can be affected by the game area, the number of players, game instructions and verbal encouragements, the number and duration of the series, the total duration of the session and the presence of goalkeepers (Dellal et al., 2008; Rampinini et al., 2007). Despite the fact that martial arts (i.e. TKD) do not have as many factors as soccer integrated training, the authors of the present study have tried to neutralize the independent variables such as the environmental conditions during training (Table 1). The influence of each session on other sessions, and chronobiological effects were controlled by the methodological counterbalanced design. Standardized verbal encouragements were used to keep the exercise intensity as high as possible through each session. One 10 s specific exercise could correspond to the duration of one long attack during competition (Bouhlel et al., 2006). The 10–20s intermittent exercise was used to mimic anaerobic and aerobic metabolism required in TKD (Bouhlel et al., 2006).

The Bandal Chagui or roundhouse kick, performed during each 10 s exercise interval, can be considered as the simplest, fastest and most often used technique in TKD competition (Falco et al., 2009). The results of the present study show that adolescent athletes can maintain high intensity (i.e. 90–95% HRmax) in TKD specific training as well as in short-duration interval running. These results are consistent with Pieter et al. (1990) and Toskovic et al. (2002). Indeed, these previous studies have suggested that kicking and punching technical combinations solicited ∼ 90% HRmax and elicited HR into cardiovascular training zones (Pieter et al., 1990; Toskovic et al., 2002). However, the cardiovascular responses to kicking in the present study were considerably higher than that reported by Bridge et al. (2007) (69.4% of HRmax) during TKD training by experienced athletes. All of these intensities fall well within the typical training session recommended guidelines identified by the American College of Sports Medicine (ACSM) for improving and maintaining cardiovascular fitness (1998). Although the current ACSM’s cardiovascular conditioning guidelines are based on studies investigating various exercise modes, however, it seems pertinent to apply these guidelines until further evidence is apparent from sport-specific conditioning studies. The only existing TKD-specific study suggested that training forms ≤10 minutes were insufficient to elicit cardiovascular adaptations (Melhim, 2001). Maintaining exercise intensity at 90–95% of HRmax using TKD specific techniques shows that this type of training design increases the HR responses to the same levels as that described for short-duration interval running. Thus, specific TKD technical interval training should allow for the development and maintenance of cardiovascular fitness at a level that is necessary for competition conditioning. Nevertheless, this has to be investigated by a longitudinal training study. In this context, not only training adaptations should be investigated alone, but also injury rate. Indeed, depending on the injury rate imposed by TKD specific training, coaches could use running training and its possible benefit to avoid the eventual injuries that may accompany TKD specific training.

In addition to the similarity of the HR responses between the two training methods, the TL calculated by Edwards’ TL (1993) and the training impulse described by Banister (1991) were not significantly different from the two modes of training. This result confirmed that practicing TKD movements during high-intensity interval training enables HR responses to increase to the same levels as running interval training.

The reported RPE between short-duration interval running and TKD specific technical interval training indicates the similarity of the training stress imposed on the athlete during these two types of training methods. These perceptions of training stress, which can include both physical and psychological stress, were similar to the typical classification identified by the American College of Sports Medicine [ACSM] (1998) for improving and maintaining cardiovascular fitness.

The results of various investigations have validated the use of RPE as a marker of training intensity during high-intensity intermittent exercise (Coutts et al., 2009; Impellizzeri et al., 2004). The increased anaerobic contribution to energy production during high-intensity interval training (Drust et al., 2000) used in the present study may explain the weak correlation between RPE and various HR methods for quantification of TL (e.g. Banister’s TRIMP [r = 0.14], and Edwards’ TL [r = 0.20]). These results are similar to previous studies examining the relationship between RPE and HR measures during intermittent exercise (Chen et al., 2002). Therefore, Chen et al. (2002) in their meta-analysis study, demonstrated that the 95% confidence interval of validity coefficients between HR and RPE was 0.40–0.62 during progressive intermittent exercise. The present results provide confirmation that session-RPE is not a valid substitute for HR measures during high-intensity interval running, or specific TKD technique interval training. In this context, other studies (Borg et al., 1985; Coutts et al., 2009) have suggested that factors other than HR can contribute to the perception of fatigue following high-intensity interval training. In this context, one limitation of the present study is that blood lactate concentration was not assessed. Consequently, the correlation between RPE and blood lactate concentration could not be determined. Indeed, various researches have shown that the combination of HR and blood lactate concentration predicts RPE more accurately than either variable taken alone (Borg et al., 1985; Coutts et al., 2009).

## Conclusion

In summary, the findings of the present study support the idea that TKD specific technical skills (e.g. Bandal Chagui) performed at a high pace enable HR responses to increase similarly to that of running during high-intensity interval training, which in turn has been shown to be effective in enhancing adolescent athletes’ endurance (Obert et al., 2001). This finding clearly suggests that specific TKD technical interval training can be used as an effective training mode to enhance aerobic fitness in TKD. Further longitudinal studies are required to compare the training effects of these two methods on the enhancement of TKD athletes’ endurance.

## Figures and Tables

**Table 1:** Methodological counterbalanced design, with the respective average ambient conditions on each of the legs of the study

		Session’s Type	Ambient conditions (°C)	Atmospheric pressure (mm hg)	Relative humidity (%)
1^st^ Group	10:00 AM	(I)	17	1018	85
		(J)	16	1011	88

2^nd^ Group	7:00 PM	(J)	15	1018	88
		(I)	15	1010	90

(I) short-duration interval running; (J) specific TKD technical interval training

**Table 2:** Characteristics of the athletes (mean = 18)

Age (Years)	Body mass (kg)	Body Height (m)	V_max_ (km × h^−1^)	HR_max_ (b × min^−1^)	Resting HR (b × min^−1^)	HR Reserve (b × min^−1^)
14 ± 2	55.2 ± 8.5	1.68 ± 0.08	19.4 ± 2.1	201 ± 6	63 ± 8	139 ± 9

Values are mean ± SD; V_max_= maximal running speed; HR_max_= maximum heart rate

**Table 3:** Comparison of HR methods and RPE recorded for the two different training methods

	**HRres (%)**	**Banister’s TRIMP (au)**	**Edwards’ TL (au)**	**RPE (au)**
	
**I**	72.88 ± 5.09	74,92 ± 10,2	118 ± 12,86	5,88 ± 2,31
**J**	71.24 ± 5.43	71,69 ± 11,2	114,31 ± 13,04	5,84 ± 1,37

Values are mean ± SD

(I) short-duration intermittent running

(J) specific TKD technical interval training (au) arbitrary unit
